# Orchid bee diversity responds positively to forest cover and landscape heterogeneity in the Brazilian Savanna

**DOI:** 10.1007/s00442-026-05920-7

**Published:** 2026-06-21

**Authors:** Fernanda G. de Sousa, Leonardo L. Bergamini, Milena F. Diniz, Paulo De M. Júnior, Rosane G. Collevatti, Daniel P. Silva

**Affiliations:** 1 Animal Biodiversity Program, Federal University of Goiás, Goiânia, 74001-970 GO Brazil; 2 Fundação Instituto Brasileiro de Geografia e Estatística (IBGE), Gerência de Meio Ambiente e Geografia, IBGE Ecological Reserve, BR-251 Highway, Km 46, Brasília, DF Brazil; 3 Center for Conservation & Sustainability, Smithsonian National Zoo and Conservation Biology Institute, MRC 705, P.O. Box 37012, Washington, DC 20013‑7012 USA; 4 The MetaLand: Theory, Metacommunity, and Landscape Ecology Lab, Ecology Department, Federal University of Goiás, Goiânia, 74001-970 GO Brazil; 5 Laboratório de Genética & Biodiversidade, Universidade Federal de Goiás, Goiânia, 74001-970 GO Brazil; 6 Conservation Biogeography and Macroecology Lab, Instituto Federal de Goiás, Anápolis, Goiás Brazil

**Keywords:** Fragmentation, Habitat loss, Pollinators, Euglossine, Brazilian Cerrado

## Abstract

Habitat loss and degradation can cause population declines in bee species, mainly through agricultural and cattle-raising pasture activities, resulting in the loss of ecosystem services. Understanding how pollinator biodiversity responds to anthropogenic changes is necessary to dimension and mitigate the impact of the human effect on the ecological and agricultural functions performed by this group. We aimed to evaluate how landscape structure (composition and configuration) influences orchid bee diversity (i.e., species richness, abundance, and species composition) in the Brazilian Cerrado Savanna. We collected the orchid bee community with chemical baits in natural patches (forest or savanna), patch edges, and matrix (agriculture or pasture) sites in 20 landscapes. We used variance inflation factor and generalized linear mixed models to evaluate the effects of landscape structure at different spatial scales on species richness, and abundance, and PERMANOVA on species composition. We sampled 728 euglossine bees from four genera and 14 species. Species richness and abundance were explained by forest cover, compositional heterogeneity, and shape of patches. Species composition was explained by forest cover. The increase in natural area and landscape diversity favored the assemblages of orchid bees. The findings of our study highlight the importance of forest cover and landscape compositional heterogeneity in supporting the species richness and abundance of orchid bees in the Brazilian Cerrado.

**Supplementary Information:**

The online version contains supplementary material available at 10.1007/s00442-026-05920-7.

## Introduction

Global land use changes have been critically affecting natural ecosystems, leading to biodiversity loss, climate change, and food security challenges (Klein et al. 2007; Lambin and Meyfroidt 2011; Grassi et al. 2017; Winkler et al. 2021). These impacts are particularly pronounced in tropical regions, which have experienced rapid deforestation and agricultural expansion over the last two decades (Winkler et al. 2021). For example, the Brazilian Cerrado, the largest and most diverse tropical savanna worldwide (Mittermeier et al. 1998), has lost approximately 50% of its original area in the last 50 years (Klink and Machado 2005; Sano et al. 2010; Alencar et al. 2020) and continues to shrink at an alarming rate (Silva and Bates 2002; Rausch et al. 2019a, b). Habitat loss and degradation influence population dynamics and species distribution across landscapes, mainly through the expansion of croplands and cattle-raising pastures (Forero-Medina and Vieira 2007). This scenario may lead to decreased biodiversity in wild populations (Brow and Paxton 2009) and to the loss of essential ecosystem services that maintain ecological balance (DeFries et al. 2004; Potts 2016).

Pollination is among the most critical ecosystem services affected by habitat modification (Potts 2016; Dicks et al. 2021). Bees are the most effective pollinators due to their coevolutionary relationships with plants, foraging behavior, pollen transport mechanisms, species diversity, and life-history traits (Kevan and Baker 1983; Ollerton 2017). As key pollinators, bees play a crucial role in food production, biodiversity, and ecosystem stability. The global decline in bee populations has led to significant reductions in plant diversity and abundance (Dirzo et al. 2014; Goulson et al. 2015; Lundgren et al. 2016). Disturbances at landscape scales, such as deforestation, fragmentation, and the conversion of natural areas, are significant for pollination systems due to their effect on pollinator density, movement, and plant demography (Hadley and Betts 2012). Therefore, understanding how pollinator biodiversity responds to anthropogenic changes at different scales is necessary to quantify and mitigate the impact of human activity on the ecological and agricultural functions (Winfree et al. 2011; Ollerton 2017).

Habitat loss and fragmentation lead to the reduction of floral and nesting resources and, therefore, have been proclaimed as the main factor responsible for the decline in bee pollinator richness and abundance (Brosi et al. 2008; Brow and Paxton 2009; Potts et al. 2010; Viana et al. 2012; Coswosk et al. 2018). These processes are deemed to cause more pronounced effects on solitary wild bees than on (eu)social ones (Steffan-Dewenter et al. 2002) and tropical regions (Ollerton et al. 2011). Solitary bees exhibit smaller population sizes than eusocial species which reduces their capacity to find floral and nesting resources; therefore, resource scarcity directly constrains their survival and reproductive success (Steffan-Dewenter et al. 2002). In contrast, social bees benefit from division of labor within the colony, which enhances foraging efficiency and allows for the more effective exploitation of dispersed resources (Steffan-Dewenter et al. 2002).

Studies evaluating the influence of landscape on bees have indicated the importance of a high proportion of natural areas for diversity (Kennedy et al. 2013; Moreira et al. 2015; Sousa et al. 2022). Moreover, high-quality habitats with surrounding high-quality land cover have also been shown to positively influence bee abundance and richness (Kennedy et al. 2013; Sousa et al. 2022). More heterogeneous landscapes provide essential resources for these pollinators, with a variety of niche availability (Viana et al. 2012; Moreira et al. 2015; Antonini et al. 2016; Boscolo et al. 2017). However, it is essential to consider landscape composition and configuration. Intense cropland activities dominated by conventional monoculture or low-diversity farming fields can negatively affect bee diversity compared to diversified and organic fields, due to low-quality habitats, and increased exposure to pesticides (Kennedy et al. 2013).

In addition to cropland cover, the influence of pasture cover (livestock grazing areas) on bee communities is also context-dependent (Aguiar et al. 2015; Carneiro et al. 2021). Although pasture cover can provide resources for habitat generalist bees, it also has negative impacts, depending on management practices (Aguiar et al. 2015). Recent studies have also highlighted the importance of landscape spatial patterns and structures (e.g., forest cover and configurational and compositional heterogeneity) in mitigating the impacts of habitat fragmentation on bee communities (Hass et al. 2018; Miljanic et al. 2018; Gutiérrez-Chacón et al. 2020). Therefore, studies testing the independent effects of landscape configuration and composition are essential to guiding conservation decisions which landscape attributes should be emphasized to ensure effective spatial designs (Hadley and Betts 2012; Betts et al. 2019).

Orchid bees (Apidae: Euglossini) have been utilized as model organisms in ecological studies on habitat disturbance for many decades due to their importance as pollinators (Powell and Powell 1987; Moura and Schlindwein 2009; Silva et al. 2015; Rosa et al. 2015; Moreira et al. 2017; Cândido et al. 2018; Sousa et al. 2022). Orchid bees are known for pollinating for hundreds of native and cultivated plant species (Dressler 1982; Gimenes 2002; Ramírez et al. 2002). Many species have considerable flying capacity and may travel long distances through continuous forests, such as *Eulaema nigrita* Lepeletier, 1841 and *Euglossa imperialis* Cockerell, 1922, which have been recorded moving several kilometers across forested landscapes (Janzen 1971; Wikelski et al. 2010; Pokorny et al. 2015).On the other hand, some other small-bodied species, including *Euglossa cordata* (Linnaeus, 1758), and *Euglossa townsendi* Cockerell, 1904, are forest-dependent and rarely leave forest fragments, showing higher sensitivity to habitat disturbance (Milet-Pinheiro and Schlindwein 2005). A multiscale approach has demonstrated that orchid bee communities respond differently to landscape composition at different on spatial scale, reinforcing the importance of evaluating landscape structure and habitat availability across multiple spatial extents (da Silva Carneiro et al. 2022). Therefore, studies that advance the knowledge of orchid bees are essential to support conservation actions involving this group and to maintain the biodiversity of various plant species (Giannini et al. 2015).

Here, we investigate how the conversion of natural areas and the resulting habitat loss and fragmentation influence orchid bee diversity in the Brazilian Cerrado. We hypothesize that reduced natural habitat cover and structural heterogeneity will negatively affect orchid bee species richness, abundance, and composition, as smaller, more isolated patches tend to harbor fewer species and restrict dispersal (Fahrig 2003).Similarly, increased agricultural and pastureland cover will reduce orchid bee diversity by reducing resource availability, increased pesticide exposure, and habitat simplification (Kennedy et al. 2013). Furthermore, we expect irregular patch shapes to intensify edge effects, further compromising nesting and foraging resources.

## Materials and methods

### Study area and sampling sites

**Fig. 1:**
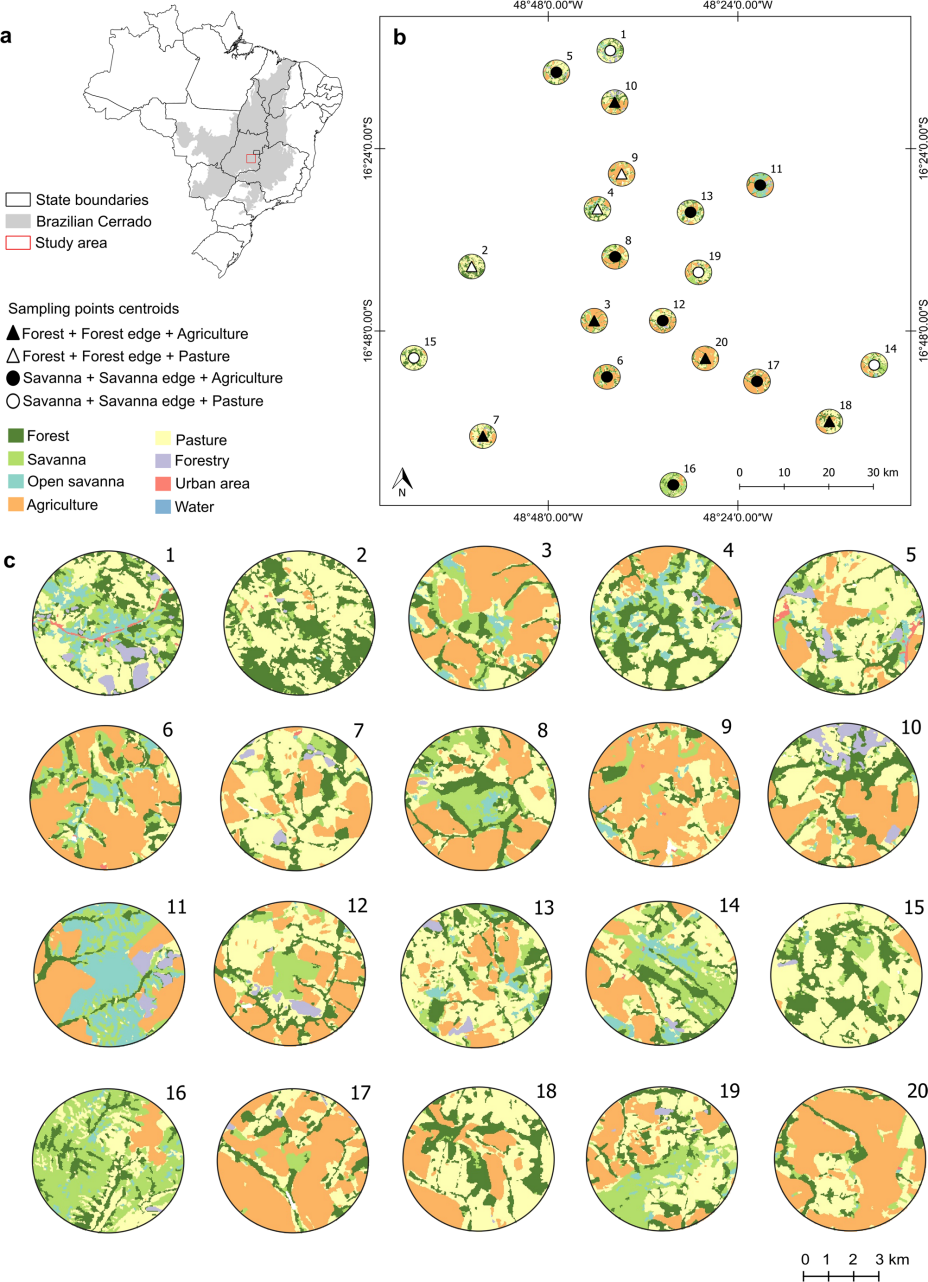
Location and composition of the study landscapes. (**a**) The Brazilian Cerrado distribution (gray) depicts the study area. (**b**) The COFA-LTER study area comprises the land cover composition of the 20 sites sampled for Euglossini bees with a buffer size of a 3 km radius. (**c**) The 20 local landscapes are detailed. Land cover colors and symbols indicate sampling centroids for distinct categories, as shown in the figure legends. Number of sampled landscapes per land-cover class: Forest: 8 landscapes; Forest edge: 8 landscapes; Savanna: 12 landscapes; Savanna edge: 12 landscapes; Pasture: 7 landscapes; Cropland: 13 landscapes

The study area is part of Agricultural Landscape Dynamics and Impacts on Biodiversity Long-Term Ecological Research (LAND-LTER; Fig. 1). We conducted this study in 20 landscapes, with a minimum distance of 10 km between them, in Central Brazil, an area covered initially by the Cerrado biome, the second largest biome in Brazil and the most biodiverse and threatened savanna in the world (Silva and Bates 2002; Mittermeier et al. 2005). The Cerrado biome is naturally composed of a mosaic of savanna-like formations and vegetation types (Ribeiro and Walter 1998). However, the landscapes of our study area are composed of a mosaic of the natural vegetation of savannas (savanna and open savanna) and forest formations (riparian and seasonally dry forests), cropland (soybean and corn), pasture, *Eucalyptus* plantation, urban area (urban and rural buildings and road), and water because of constant deforestation expansion at high rates yearly (Alencar et al. 2020). In our study area, considering a landscape delimited by a 3 km radius, pastures, and croplands are the predominant land-use types, covering 33.08% and 29.11% of the occupation, respectively), followed by forest (17.68%), savannas (13.11%), open savanna (4.77%), forestry (1.92%), and urban areas (0.26%).

### Bee community sampling

We selected natural patches (forest or savanna), patch edges, and matrix (agriculture or pasture) sites to sample the orchid bee community in each landscape. We collected these bees in four combinations among the 20 landscapes: (i) forest + edge forest + agriculture (FEA; five landscapes); (ii) forest + edge forest + pasture (FEP; three landscapes); (iii) savanna + savanna edge + agriculture (SEA; eight landscapes); and (iv) savanna + edge savanna + pasture (SEP; four landscapes). We installed two Euglossini sampling stations at a minimum distance of 50 m apart from each other at each site, totaling six sampling stations for the landscape. Each sampling station comprised six bait (e.g., eucalyptol, methyl salicylate, vanillin, eugenol, methyl cinnamate, and benzyl acetate) soaked in cotton, installed 1.5 m above ground and 3 m apart from one another. We built the traps from plastic polyethylene terephthalate bottles with three funnel inlets on their sides, and sandpaper to increase friction at the entrance. The minimum distance of savanna/forest interior stations from the fragment edge was 100 m, the edge stations installed in the edge from the interior were 2–10 m, and the agriculture/pasture stations from the fragment edge were 100 m. We placed bait traps for 48 h during the rainy season (January to March 2022) due to the high activity of euglossine bees during this season (Roubik and Hanson 2004). We identified the bees at the species level following the accepted names from the Catalogue of Bees (Hymenoptera, Apoidea) in the Neotropical Region (Rebêlo and Moure 1995; Roubik 2004; Bembé 2007; Ferrari and Melo 2014; Hinojosa-Díaz and Engel 2014; Nemésio 2009; Moure and Melo 2022). The specimens were deposited in the Zoology Collection of the Universidade Federal de Goiás (Goiânia, Goiás, Brazil) and the Zoology Collection of the Instituto Brasileiro de Geografia e Estatística (IBGE; Brasília, Distrito Federal, Brazil).

### Landscape metrics

We performed spatial analyses considering the landscapes delineated from the point centroids of natural site sampling to measure the influence of landscape structure on orchid bees. We used a land cover map at a resolution of 30 m from the Brazilian Annual Land Use and Land Cover Mapping Project [collection 7, 2021] (MapBiomas; http://www.mapbiomas.org), corresponding to the map available for the year when the field survey took place (2021). The land cover maps of this platform were derived from Landsat-like image classification. The map has the land cover types: forest, savanna, open savanna, agriculture, pasture, forestry, mining, urban area, and water (lake or river).

We used a multiscale approach because species have different daily movement distances and dispersal capacities. First, we delimited buffers of 0.5, 1, 1.5, 2, and 3 km in radius from the centroids of the sample point to delimit landscapes. We used these scales considering the flight range of the orchid bees among the diversity of the group (Borges et al. 2020) and the great responses in other studies, with ranges of the scales between 0.5 km and 3 km (da Silva Carneiro et al. 2022; Sousa et al. 2022). We calculated the landscape metrics for each scale. We calculated forest cover (%FC), savanna cover (%SC), agriculture cover (%AC), pasture cover (%PC), and compositional heterogeneity (CH) to measure the influence of landscape composition on orchid bees. We calculated forest cover (%FC) as the percentage of the natural vegetation of riparian and seasonal forests and savanna cover (%SC) as the percentage of savanna and open savanna. We calculated landscape compositional heterogeneity using the Shannon landscape diversity index (SHDI) (McGarigal et al. 2012). We calculate the proportion of all land cover type classes in the landscapes. These landscape metrics were chosen because they are commonly used indicators of habitat loss (Fahrig 2013, 2017; McGarigal et al. 2012). However, the decrease in habitat amount also implies a negative effect of habitat fragmentation on species richness and occurrence, as noted by Saura (2020).

We calculated the mean shape index (SH) to measure the influence of the configuration of the landscape on orchid bees. We calculated the SH as the complexity of each patch shape, including forest and savanna patches. The SH equals 1 if all patches are square (assuming the patch is regular) and increases as the shapes of patches become more complex (Patton 1975; McGarigal et al. 2012). We calculated the landscape metrics using the *landscapemetrics* R package (Hesselbarth et al. 2019).

### Data analyses

We calculated the estimated species richness, abundance, and species composition as response variables (Table S1). We assessed species richness using the Jackknife method (Heltshe and Forrester 1983). The first-order Jackknife estimator is an incidence-based, nonparametric method used to estimate total species richness, reducing bias associated with sampling effort and rare species (Heltshe and Forrester 1983). To evaluate differences in bee species composition among landscapes, we constructed a similarity matrix using the Jaccard similarity index (Chao et al. 2005), which is based on species presence–absence data. Low Jaccard similarity values indicate high dissimilarity in species composition between pairs of landscapes. We calculated the estimated species richness and species composition using the ‘specnumber’ and ‘vegdist’ functions of the *vegan* R package. We evaluated spatial autocorrelation in our community data (estimated species richness and abundance) using Moran’s I test and the *ape* R package (Paradis and Schliep 2019). Assessing spatial autocorrelation allows testing of whether nearby sampling sites are non-independent, which could bias statistical inference. We used the Mantel test to evaluate spatial autocorrelation in species composition across landscapes using the ‘mantel’ function with the *ape* R package (Paradis and Schliep 2019).

**Table 1:** The abundance of Euglossini bee species by land cover class sampling

Species	Forest	Forest Edge	Savanna	Savanna Edge	Pasture	Agriculture	Total
*Aglae caerulea* Lepeletier & Serville, 1825	0	0	2	0	0	0	**2**
*Eufriesea auriceps* (Friese, 1899)	4	5	21	20	6	20	**76**
*Eufriesea* cf. *mussitans* (Fabricius, 1787)	1	0	0	0	0	0	**1**
*Euglossa azurea* Ducke, 1902	0	1	0	2	0	0	**3**
*Euglossa cordata* (Linnaeus, 1758)	0	0	0	0	1	1	**2**
*Euglossa imperialis* Cockerell, 1922	20	14	41	14	2	1	**92**
*Euglossa leucotricha* Rebêlo & Moure, 1996	0	0	1	0	1	1	**3**
*Euglossa melanotricha* Moure, 1967	1	0	1	2	1	4	**9**
*Euglossa pleosticta* Dressler 1982	0	0	0	1	1	0	**2**
*Euglossa securigera* Dressler 1982	2	1	7	7	1	7	**25**
*Euglossa townsendi* Cockerell, 1904	6	1	10	4	10	14	**45**
*Euglossa truncata* Rebêlo & Moure, 1996	1	1	2	4	1	4	**13**
*Eulaema cingulata* (Fabricius, 1804)	6	6	24	27	6	14	**83**
*Eulaema nigrita* Lepeletier, 1841	52	24	94	55	53	94	**372**
**Total**	**93**	**53**	**203**	**136**	**83**	**160**	**728**

We selected the scale of effect for each response variable (species richness, abundance, and species composition) and the landscape metrics for the models. We used R^2^ (coefficient of determination), which represents the proportion of variance in the response variable explained by the predictor, to quantify the strength of the relationship between species richness, abundance, and species composition, and the landscape structure to choose the best scale of effect, as proposed by Jackson and Fahrig (2015; Supplementary Table S4). As a descriptor of species composition in this analysis, we used reduced ordination using principal coordinates analysis (PCoA) based on abundance data and the Jaccard similarity index. We performed a multiscale test of independence for multivariate vectors using the *multifit* R function (Huais 2018) to select the scale of effect. Then, we estimated the variance inflation factor (VIF) to assess collinearity among landscape metrics in all models (Dormann et al. 2013) and to eliminate variables that inflate the models of species richness and abundance. In our study, we set a threshold of < 3 for VIF values to indicate variance inflation (Zuur et al. 2009). We assumed that VIF values below this threshold indicate the independence of the predictor variables, and high VIF values (> 3) indicate that the predictor variables are highly correlated with each other. We used the function ‘summ’ of the *jtools* R package to calculate the VIF (Long 2022, 2024). We removed crop land cover in the models with species richness as response variables and pasture cover in the models with abundance as response variables that were high levels of collinearity (VIF > 3, see Supplementary Table S5). Regarding the species composition, the variables selected were tested for collinearity using Pearson’s correlation, and the variable pasture cover was removed (*r* > 0.7). Afterward, the scales were standardized using z-score (Cohen et al. 2003).

After we excluded landscape metrics with VIF > 3, we used generalized linear mixed models (GLMM) to evaluate the effects of landscape metrics on estimated species richness and abundance. We used the combinations of landscapes (FEA, FEP, SEA, and SEP) as a random effect. After verifying all assumptions, we used the Gaussian distribution for all models. For this, we used the *lme4* package (Bates et al. 2015). Model selection was performed using the ‘dredge’ function in the *MuMin* R package (Barton 2015), which builds models for all possible combinations of variables. We selected the best model using Akaike’s information criterion corrected for small sample sizes (AICc; Burnham and Andersen 2002). We selected models with ΔAICc < 2.0 (Zuur et al. 2009), and then we built model averages using the ‘model.avg’ function (Burnham and Andersen 2002).

We performed Permutational Multivariate Analysis (PERMANOVA) with 999 permutations to analyze the habitat loss and fragmentation on species composition, using the Jaccard similarity index, using the ‘adonis2’ function in the *vegan* package (Oksanen et al. 2017). The multidimensional composition of orchid bee species across the entire landscape was plotted using the first axis of a PCoA based on abundance data and the Jaccard similarity index, along with landscape metrics that had a significant p-value (*p* < 0.05) in the PERMANOVA analysis. All analyses were performed in R (R Core Team 2024).

## Results

We sampled 728 Euglossini bees from four genera and 14 species. *Eulaema nigrita* Lepeletier, 1841, was the most abundant species (*n* = 372), followed by *Euglossa imperialis* Cockerell, 1922 (*n* = 92), and *Eulaema cingulata* (Fabricius, 1804) (*n* = 83). Three species, *Eufriesea* cf. *mussitans* (Fabricius, 1787), *Euglossa pleosticta* Dressler 1982; and *Euglossa truncata* Rebêlo & Moure, 1996, were registered for the first time in the state of Goiás (Brazil). The estimated species richness ranged from 1 to 14 and abundance from 10 to 64 (Supplementary Table S1 and S2).

Across sampling sites, landscapes consisted of a heterogeneous mosaic of forest and savanna formations interspersed with anthropogenic land uses, including pasture lands, agricultural fields (mainly soybean and maize), silviculture plantations, urban areas, and water bodies. Pasture was the dominant land-use type, representing 33.1% of total land cover, followed by agriculture (29.11%) and forest cover (17.68%) within a 3 km radius, reflecting the strong anthropogenic influence across the study region. Savanna formations accounted for only 4.77% of landscape cover, while silviculture plantations and urban areas accounted for 1.92% and 0.26%, respectively. Habitat configuration also showed substantial variation among sites.

Species richness showed a scale of effect at 3 km for FC and CH, and at 0.5 km for SH, whereas abundance responded at 3 km for FC and at 1.5 km for both CH and SH. Species composition was influenced by FC at 1.5 km. We found no significant spatial autocorrelation for estimated species richness (*r* = − 0.05; *p* = 0.50), abundance (*r* = − 0.05; *p* = 0.50; Supplementary Table S3), or species composition (*r* = − 0.08; *p* = 0.45).

**Fig. 2:**
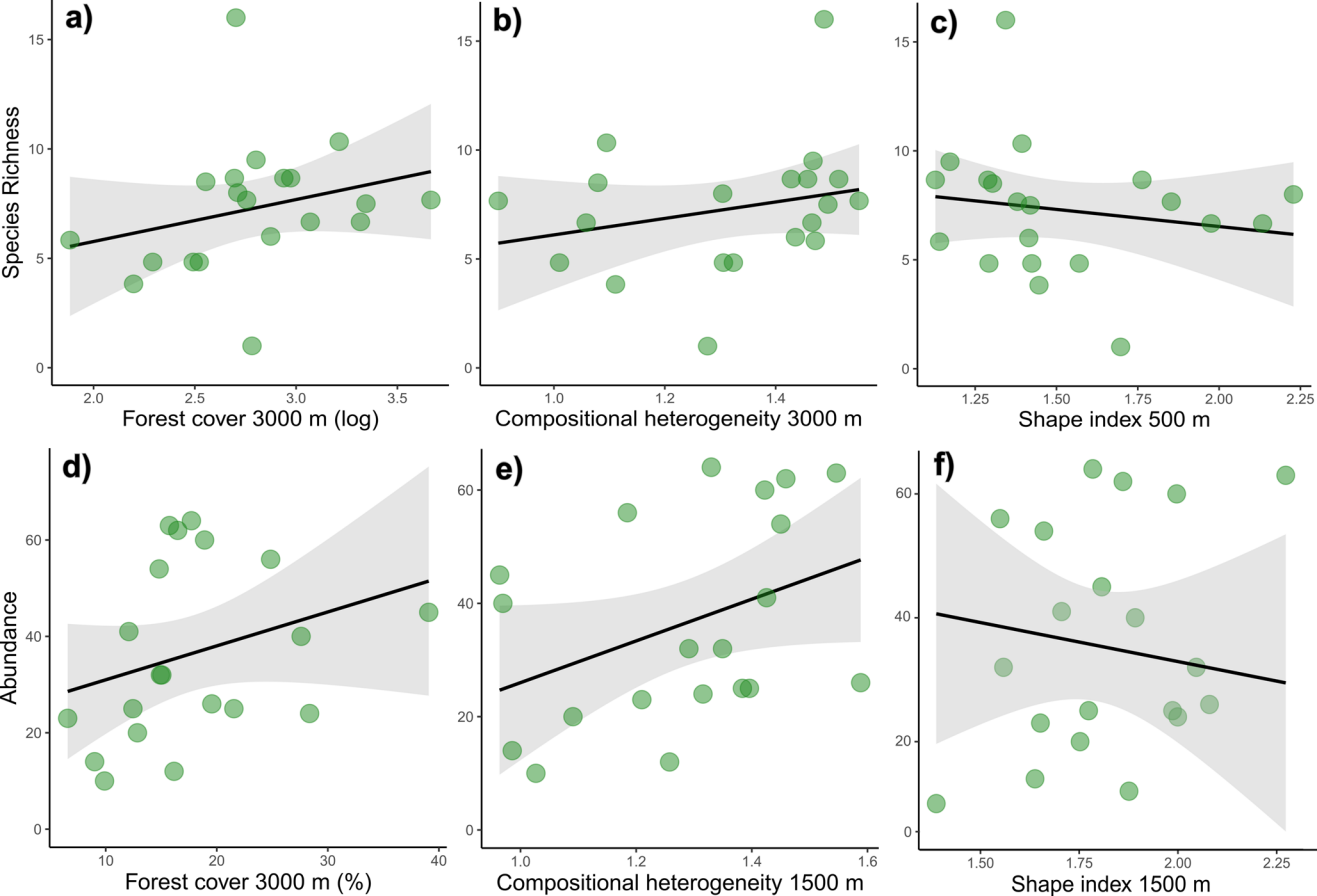
Relationship between Euglossini species richness and abundance, and forest cover (**a**,** d**), compositional heterogeneity (**b**,** e**), and shape index (**c**,** f**), based on sites in the Brazilian Cerrado. The shaded areas in the panel show 95% confidence intervals

CH was the strongest predictor of species richness and was included in four of the five selected models (Table 2; Fig. 2b). FC and SH were also selected in three models, each with low AIC (Table 2; Fig. 2a and c). CH was also the strongest predictor of abundance and was included in two of the selected models with low AIC (Table 2; Fig. 2e). SH and FC were also selected (Table 2; Fig. 2d and f).

**Table 2:** Selected models (< 2ΔAICc from the best model) of Euglossini bees in 20 landscapes in the Brazilian Cerrado. Predictors include estimated species richness and abundance. Weight (Akaike’s weight of evidence) was corrected for the sample size and the number of model parameters. ∆AICc corrects for small sample sizes by accounting for the difference between each model and the best model

	Intercept	Forest Cover	Compositional Heterogeneity	Shape Index	df	AICc	ΔAICc	Weight
Estimated Species Richness	−2.47	2.68	4.41	−2.32	6	102.5	0.0	0.23
	−4.68	2.39	3.96		5	102.8	0.29	0.20
	4.70		3.71	−1.51	5	103.7	1.19	0.12
	2.32		3.77		4	103.9	1.44	0.11
	2.85	2.66		−2.07	5	104.0	1.48	0.11
Model average	**−0.42**	**2.57**	**4.04**	−**2.04**				
Abundance	−8.61		37.08	−1.40	5	162.0	0.00	0.46
	−26.15	0.94	45.63	−7.04	6	162.4	0.43	0.37
Model average	**−16.43**	**0.94**	**40.88**	**−3.92**				

df, degree of freedom

**Fig. 3:**
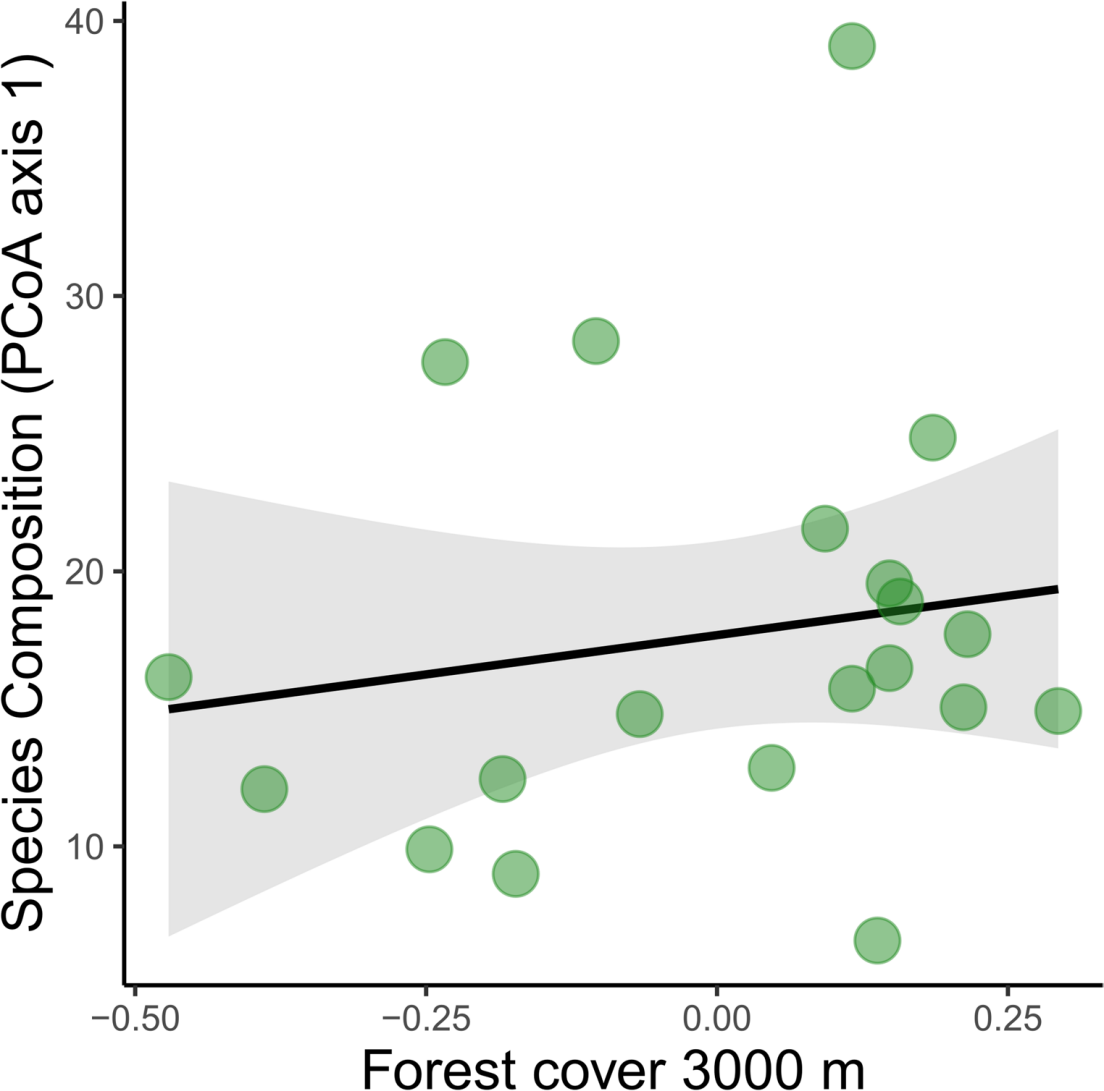
Relationship between Euglossini species composition and forest cover based on sites in the Brazilian Cerrado. The shaded areas in the panel show 95% confidence intervals

The first PCoA axis, used as a proxy for species composition, explained 28% of the variation in orchid bees species composition among landscapes. This axis represents changes in community structure associated with landscape, separating sites dominated by disturbance-tolerant species from those harboring more forest-associated species. FC explained variation in species composition (Table 3; Fig. 3; R^2^ = 0.11, F = 2.33, df = 1, *p* = 0.01), although the relatively low R² value suggests that additional environmental or spatial factors may also contribute to community variation.

**Table 3:** Euglossini bee compositional differences along landscape variations in the Brazilian Savanna. Permutational multivariate analysis

	*R* ^2^	df	F	*p*
Forest Cover	0.11	1	2.46	**< 0.01**
Savanna Cover	0.05	1	1.18	0.27
Agriculture Cover	0.06	1	1.14	0.17
Compositional Heterogeneity	0.06	1	1.45	0.15
Shape Index	0.04	1	0.90	0.54
Residuals	0.65	14		

R^2^, coefficient of determination for PERMANOVA; df, degree of freedom; F, ratio of variations. Bold values mean p-value < 0.05

## Discussion

Our results indicate that landscape composition and configuration influence the species richness, abundance, and species composition of orchid bees in the Brazilian Savanna. CH and FC positively affected species richness at large spatial scales (3 km each) and abundance at large (3 km) and medium (1.5 km) scales, respectively. This suggests that the surrounding landscape influences orchid bees at different spatial scale, confirming our result that the SH also influenced the species richness and abundance of bees at small and medium scales (0.5 km and 1.5 km) scales. Additionally, species composition was strongly affected by FC at the 3 km scale.

Forest cover had a positive effect on the species richness, abundance, and species composition of orchid bees, indicating that large areas of forested land contribute to higher species richness and abundance of orchid bees. Other studies have reported similar findings in savanna landscapes (da Silva Carneiro et al. 2022; Sousa et al. 2022), reinforcing the importance of the natural cover area in sustaining species populations (Fahrig 2013). The amount of available habitat directly affects the resources on which bees depend, including floral resources (pollen sources and nectar), nesting substrate, and material for nest building (Roubik and Hanson 2004). Our results indicate a substantial decline in bee populations in landscapes with low percentages of natural habitat cover, as only a small subset of euglossine species can colonize and survive in highly disturbed environments. Although these bees are strongly associated with forest habitats (Dressler 1982), species perceive differently to environmental conditions based on their degrees of specialization. This result may explain the increase in orchid bees in agroecosystems with high compositional heterogeneity (Briggs et al. 2013; Moreira et al. 2017; Sousa et al. 2022).

Compositional heterogeneity positively affected the richness and abundance of orchid bees, showing that more heterogeneous landscapes favor bee communities. This pattern can be attributed to the variety of land-use types in the study area, and the intrinsic diversity of Cerrado vegetation. The Cerrado is characterized by a mosaic of distinct forest formations that provide a wide range of floral and nesting resources across vegetation types. Such high habitat heterogeneity supports both species strongly associated with closed-canopy habitats (e.g., forested areas) and generalist species adapted to open savanna environments. Forested habitats, including gallery forests and seasonal semideciduous forests, also function as dispersal corridors for orchid bees originating from adjacent rainforest regions (Moura and Schlindwein 2009; Silva et al. 2013). While euglossine bees generally exhibit higher diversity in rainforest ecosystems than other South American biomes (Nemésio and Silveira 2007), evidence suggests that species occurring in savannas tend to be more generalist, exploiting a broader range of habitats and floral resources (Juncá et al. 2005; Sousa et al. 2022).

Generalist euglossines such as *Eulaema nigrita* and *Euglossa imperialis* increase their abundance in disturbed environments, demonstrating resilience to landscape changes (Powell and Powell 1987; Silva and De Marco 2014) and adaptation to ecotone areas (Rosa et al. 2016). Nonetheless, most studies have shown negative environmental impacts on orchid bee diversity due to habitat fragmentation and degradation (Powell and Powell 1987; Brown 1992; Brosi et al. 2008; Brosi 2009; Moreira et al. 2017; Storck-Tonon and Peres 2017). These generalist bees have a larger body and greater flying capability (Janzen 1971; Milet-Pinheiro and Schlindwein 2005; Wikelski et al. 2010; Pokorny et al. 2015) than small-bodied species, which may explain the dominance of *Eulaema nigrita* across all the landscapes in our study.

The landscape shape complexity (shape index) negatively affected richness and abundance of orchid bees. Landscapes with more irregularly shaped patches are often associated with higher levels of fragmentation, increased edge density, and reduced core habitat availability (Fahrig 2013). Such conditions can limit the availability of continuous foraging areas and nesting resources, which are essential for sustaining diverse and abundant bee communities. Enhanced edge effects may further reduce habitat quality and connectivity, thereby constraining population persistence (Betts et al. 2019). Although heterogeneous landscapes may increase foraging opportunities (Hipólito et al. 2018), the surrounding matrix at habitat edges can act as a barrier, restricting both resource use and movement between patches (Moreira et al. 2017). Extreme environmental conditions, such as elevated temperatures and low humidity, may challenge euglossine bees by making it hard for them to build and establish nests and fly between habitat patches in search of floral and nesting resources (Vilhena et al. 2017). This pattern suggests that maintaining more compact and less irregular habitat patches may be critical to mitigating the negative consequences of fragmentation on pollinator communities in agricultural landscapes.

In conclusion, our findings highlight the importance of forest cover and landscape heterogeneity in maintaining orchid bee species richness and abundance in the Brazilian Cerrado. These results underscore the ecological significance of preserving forested areas within the landscape to sustain the orchid bee population and, by extension, the broader ecosystem. From a conservation perspective, adopting sustainable agricultural practices and preserving forest fragments on rural properties, could mitigate the negative impacts of habitat conversion. Finally, understanding the implications of landscape composition for these key pollinators can provide valuable insights for conservation efforts and sustainable land-use management in the region.

## Supplementary Information

Below is the link to the electronic supplementary material.

Supplementary Material 1

## Data Availability

All data generated during the field samplings analyzed during this study are included in this published article (and its supplementary information files).

## Code availability

The code used for the analyses is available from the corresponding author upon reasonable request.

## References

[CR1] Aguiar AP, Carneiro GM, Araujo GP (2015) Landscape structure and species diversity in a fragmented Brazilian Amazon Forest. Landscape Ecol 30(6):1079–1090

[CR2] Alencar A, Shimbo JZ, Lenti F et al (2020) Mapping three decades of changes in the Brazilian savanna native vegetation using Landsat data processed in the Google Earth engine platform. Remote Sens 12(6). 10.3390/rs12060924

[CR107] Antonini Y, Silveira RA, Oliveira M, Martins C, Oliveira R (2016) Orchid bee fauna responds to habitat complexity on a savanna area (Cerrado) in Brazil. Sociobiology 63(2):819–825. 10.13102/sociobiology.v63i2.1038

[CR160] Bates D, Mächler M, Bolker B, Walker S (2015) Fitting Linear Mixed-Effects Models Using lme4. Journal of Statistical Software 67(1):1–48. 10.18637/jss.v067.i01

[CR3] Bembé B (2007) The influence of habitat fragmentation on the biodiversity of tropical forest fragments. Landscape Ecol 22(3):487–498

[CR4] Betts MG, Hadley AS, Kormann U (2019) The landscape ecology of pollination. Landscape Ecol 34:961–966

[CR5] Borges RC, Padovani K, Imperatriz-Fonseca VL, Giannini TC (2020) A dataset of multi-functional ecological traits of Brazilian bees. Sci Data 7(1):1–9. 10.1038/s41597-020-0461-3 32286316 10.1038/s41597-020-0461-3 PMC7156730

[CR6] Boscolo D, Tokumoto PM, Ferreira PA, Ribeiro JW, Santos JS (2017) Positive responses of flower-visiting bees to landscape heterogeneity depend on functional connectivity levels. Perspective Ecol Conserv 15(1):18–24

[CR7] Briggs HM, Perfecto I, Brosi BJ (2013) The Role of the Agricultural Matrix: Coffee Management and Euglossine Bee (Hymenoptera: Apidae: Euglossini) Communities in Southern Mexico. Environ Entomol 42(6):1210–1217 24128972 10.1603/EN13087

[CR8] Brosi BJ (2009) The effects of forest fragmentation on euglossine bee communities (Hymenoptera: Apidae: Euglossini). Biol Conserv 142(2):414–423

[CR9] Brosi BJ, Daily GC, Shih TM, Oviedo F, Durán G (2008) The effects of forest fragmentation on bee communities in tropical countryside. J Appl Ecol 45:773–783

[CR10] Brow M, Paxton RJ (2009) Effects of habitat fragmentation on pollinator communities in temperate regions. Landscape Ecol 24(9):1269–1281

[CR11] Brown BV (1992) The role of insect pollinators in tropical forests. Landscape Ecol 15(3):225–232

[CR12] Burnham KP, Andersen DR (2002) Model selection and multimodel inference: A practical information-theoretic approach. Springer

[CR13] Cândido MEMB, Morato EF, Storck-Tonon D et al (2018) Effects of fragments and landscape characteristics on the orchid bee richness (Apidae: Euglossini) in an urban matrix, southwestern Amazon. J Insect Conserv 22:475–486

[CR14] Carneiro LS, Aguiar WM, Priante CF et al (2021) The Interplay Between Thematic Resolution, Forest Cover, and Heterogeneity for Explaining Euglossini Bees Communit in an Agricultural Landscape. Front Ecol Evol 9:1–13

[CR15] Chao A, Chazdon RL, Colwell RK, Shen T (2005) A new statistical approach for assessing similarity of species composition with incidence and abundance data. Ecol Lett 8(8):148–159

[CR16] Coswosk JA, Ferreira RA, Soares EDG et al (2018) Responses of Euglossine Bees (Hymenoptera, Apidae, Euglossina) to an Edge-Forest Gradient in a Large Tabuleiro Forest Remnant in Eastern Brazil. Neotropic Entomol 47(4):447–456 10.1007/s13744-017-0533-z 28540533

[CR110] da Silva Carneiro L, Ribeiro MC, Aguiar WM de, de Fátima Priante C, Frantine-Silva W, Gaglianone MC (2022) Orchid bees respond to landscape composition differently depending on the multiscale approach. Landsc Ecol 37(6):1587–601. 10.1007/s10980-022-01442-8

[CR17] DeFries RS, Foley JA, Asner GP (2004) Land-use choices: balancing human needs and ecosystem function. Front Ecol Environ 2(5):249–257

[CR18] Dicks LV, Breeze TD, Ngo HT et al (2021) A global-scale expert assessment of drivers and risks associated with pollinator decline. Nat Ecol Evol 5(10):1453–1461 34400826 10.1038/s41559-021-01534-9

[CR19] Dirzo R, Young HS, Galetti M et al (2014) Defaunation in the Anthropocene. Science 345(6195):401–406 25061202 10.1126/science.1251817

[CR20] Dormann CF, Elith J, Bacher S et al (2013) Collinearity: a review of methods to deal with it and a simulation study evaluating their performance. Ecography 36:27–46

[CR21] Dressler RL (1982) The ecological consequences of fragmentation in tropical rainforests. Ecol Monogr 52(2):175–200

[CR120] Fahrig L (2003) Effects of Habitat Fragmentation on Biodiversity. Annual Review of Ecology, Evolution, and Systematics 34:487–515. 10.1146/annurev.ecolsys.34.011802.132419

[CR22] Fahrig L (2013) Rethinking patch size and isolation effects: the habitat amount hypothesis. J Biogeogr 40:1649–1663

[CR23] Fahrig L (2017) Ecological Responses to Habitat Fragmentation Per Se. Annual Review of Ecology, Evolution, and Systematics, 48:1–23

[CR24] Ferrari RR, Melo GAR (2014) Deceiving colors: recognition of color morphs as separate species in orchid bees is not supported by molecular evidence. Apidologie 45:641–652

[CR25] Forero-Medina GA, Vieira MV (2007) Conectividade funcional e a importância da interação organismo-paisagem. Oecologia Bras 11:493–502

[CR26] Giannini TC, Cordeiro GD, Freitas BM, Saraiva AM, Imperatriz-Fonseca VL (2015) The dependence of crops for pollinators and the economic value of pollination in Brazil. J Econ Entomol 108(3):849–857 26470203 10.1093/jee/tov093

[CR27] Gimenes M (2002) Interactions between bees and Ludwigia elegans (Camb.) Hara 103 (Onagraceae) flowers at different altitudes in São Paulo, Brazil. Revista Brasileira de Zoologia 104(19):681–689

[CR28] Goulson D, Nicholls E, Botías C et al (2015) Bee declines driven by combined stress from parasities, pesticides, and lack of flowers. Science 347(6229):1255957 25721506 10.1126/science.1255957

[CR29] Grassi G, House JI, Dentener F et al (2017) The key role of forests in meeting climate targets requires science for credible mitigation. Nat Clim Change 7:220–226

[CR30] Gutiérrez-Chacón C, Valderrama-A C, Klein A-M (2020) Biological corridors as important habitat structures for maintaning bees in a tropical fragmented landscape. J Insect Conserv 24(1):187–197. 10.1007/s10841-019-00205-2

[CR31] Hadley AS, Betts MG (2012) The effects of landscape fragmentation on pollination dynamics: absence of evidence not evidence of absence. Biol Rev 87:526–544 22098594 10.1111/j.1469-185X.2011.00205.x

[CR108] Hass AL, Kormann UG, Tscharntke T, Clough Y, Baillod AB, Sirami C, Fahrig L, Martin JL, Baudry J, Bertrand C, Bosch J, Brotons L, Burel F, Georges R, Giralt D, Marcos-García MÁ, Ricarte A, Siriwardena G, Batáry P (2018) Landscape configurational heterogeneity by small-scale agriculture, not crop diversity, maintains pollinators and plant reproduction in western Europe. Proc Biol Sci 285(1872):20172242. 10.1098/rspb.2017.2242 10.1098/rspb.2017.2242 PMC5829195 29445017

[CR150] Heltshe JF, Forrester NE (1983) Estimating Species Richness Using the Jackknife Procedure. Biometrics 39(1):1–11. 10.2307/2530802 6871338

[CR32] Hesselbarth MHK, Sciaini M, With KA et al (2019) *landscapemetrics*: an open-source R tool to calculate landscape metrics. Ecography 42(10):1648–1657

[CR125] Hinojosa-Díaz I, Engel M (2014) Revision of the orchid bee subgenus Euglossella (Hymenoptera: Apidae), part II: The viridis and mandibularis species groups. Journal of Melittology 10.17161/jom.v0i36.4777

[CR140] Hipólito J, Boscolo D, Viana BF (2018) Landscape and crop management strategies to conserve pollination services and increase yields in tropical coffee farms.Agriculture, Ecosystems & Environment 256:218–225. 10.1016/j.agee.2017.09.038

[CR152] Huais PY (2018) multifit: an R function for multi-scale analysis in landscape ecology. Landscape Ecol 33:1023–1028. 10.1007/s10980-018-0657-5

[CR34] Jackson DA, Fahrig L (2015) The role of landscape configuration in conservation planning. Landscape Ecol 30(6):975–988

[CR35] Janzen DH (1971) Euglossine bees as long-distance pollinators of tropical plants. Science 171(3976):203–205 17751330 10.1126/science.171.3967.203

[CR139] Juncá FA, Funch L, Rocha W (Eds) (2005) Biodiversidade e conservação da Chapada Diamantina. Brasília, DF: Ministério do Meio Ambiente. 411 p. ISBN 85-87166-78-6.

[CR36] Kennedy CM, Lonsdorf E, Neel MC et al (2013) A global quantitative synthesis of local and landscape effects on wild bee pollinators in agroecosystems. Ecol Lett 16:584–599 23489285 10.1111/ele.12082

[CR101] Kevan PG, Baker HG (1983) Insects as flower visitors and pollinators. Annual Review of Entomology 28:407–453. 10.1146/annurev.en.28.010183.002203

[CR100] Klein AM, Vaissière BE, Cane JH, Steffan-Dewenter I, Cunningham SA, Kremen C, Tscharntke T (2007) Importance of pollinators in changing landscapes for world crops. Proc Biol Sci 274(1608):303-13. 10.1098/rspb.2006.3721 10.1098/rspb.2006.3721 PMC1702377 17164193

[CR37] Klink CA, Machado RB (2005) A conservação do Cerrado brasileiro. Megadiversidade 1(1):147–155

[CR38] Lambin EF, Meyfroidt P (2011) Global land use change, economic globalization, and the looming land scarcity. Proceedings of the National Academy of Sciences, 108:3465–3472 10.1073/pnas.1100480108 PMC3048112 21321211

[CR39] Long JA (2022) *jtools*: Analysis and Presentation of Social Scientific Data. R package version 2.2.0. https://cran.r-project.org/package=jtools

[CR40] Long JA (2024) Tools for summarizing and visualizing regression *models*. https://jtools.jacob-long.com/articles/summ.html

[CR41] Lundgren R, Totland O, Lázaro A (2016) Experimental simulation of pollinator decline causes community-wide reductions in seedling diversity and abundance. Ecology 97(6):1420–1430 27459773 10.1890/15-0787.1

[CR42] McGarigal K, Cushman SA, Ene E (2012) FRAGSTATS v4: spatial pattern analysis program for categorical and continuous maps. Computer software program produced by the authors at. the University of Massachusetts, Amherst

[CR43] Milet-Pinheiro P, Schlindwein C (2005) Do euglossine males (Apidae, Euglossini) leave tropical rainforest to collect fragrances in sugar cane monocultures? Revista Brasileira de Zoologia 22(4):853–858

[CR44] Mittermeier RA, Myers N, Thomsen JB et al (1998) Biodiversity Hotspots and Major Tropical Wilderness Areas: Approaches to Setting Conservation Priorities. Conserv Biol 12(3):516–520

[CR121] Mittermeier RA, Gil PR, Hoffman M, Pilgrim J, Brooks T, Mittermeier CG, Lamoreux J, da Fonseca GAB (2005) Hotspots Revisited: Earth’s Biologically Richest and Most Endangered Terrestrial Ecoregions. Conservation International, Washington, D.C.

[CR45] Moreira EF, Boscolo D, Viana BF (2015) Spatial Heterogeneity Regulates Plant-Pollinator Networks across Multiple Landscape Scales. PLoS ONE 10(4):e0123628 25856293 10.1371/journal.pone.0123628 PMC4391788

[CR46] Moreira EF, Santos RLS, Silveira MS et al (2017) Influence of landscape structure on Euglossini composition in open vegetation environments. Biota Neotrop 17(1):e20160294

[CR47] Moura MO, Schlindwein C (2009) Pollinator networks in fragmented ecosystems. Oecologia 161(2):347–356

[CR48] Moure JS, Melo GAR (2022) Euglossini Latreille, 1802. In Moure JS, Urban D & Melo GER (Orgs). Catalogue of Bees (Hymenoptera, Apoidea) in the Neotropical Region – online version. Availabe at https://www.moure.cria.org.br/catalogue

[CR49] Nemésio A (2009) Bee diversity and ecology in the Brazilian Atlantic Forest. Ecol Entomol 34(4):405–413

[CR50] Nemésio A, Silveira FA (2007) Patterns of pollinator diversity in Brazilian ecosystems. Biol Conserv 137(4):512–520

[CR51] Oksanen FJ, Blanchet G, Friendly M et al (2017) Vegan: Community Ecology Package. R package version 2.4-4

[CR52] Ollerton J (2017) Pollinator Diversity: Distribution, Ecological Function, and Conservation. Annu Rev Ecol Syst 48:353–376

[CR53] Ollerton J, Winfree R, Tarrant S (2011) How many flowering plants are pollinated by animals? Oikos 120(3):321–326

[CR54] Paradis E, Schliep K (2019) ape 5.0: an environment for modern phylogenetics and evolutionary analyses in R. Bioinformatics 35:526–528 30016406 10.1093/bioinformatics/bty633

[CR55] Patton DR (1975) The role of corridors in wildlife conservation. J Wildl Manage 39(4):431–436

[CR56] Pokorny T, Loose D, Dyker G, Quezada-Euán JG, Eltz T (2015) Dispersal ability of male orchid bees and direct evidence for long-range flights. Apidologie 46:224–237

[CR105] Potts SG, Biesmeijer JC, Kremen C, Neumann P, Schweiger O, Kunin WE (2010) Global pollinator declines: trends, impacts and drivers. Trends in Ecology & Evolution 25(6):345–353. 10.1016/j.tree.2010.01.007 10.1016/j.tree.2010.01.007 20188434

[CR57] Powell SF, Powell AL (1987) Population Dynamics of Male Euglossine Bees in Amazon Forest Fragments. Biotropica 19(2):176–179

[CR58] R Core Team (2024) R: A language and environment for statistical computing. R Foundation for Statistical Computing, Vienna, Austria

[CR59] Ramírez S, Ospina M, Dressler R (2002) Abejas euglosinas (Hymenoptera: Apidae) de la Región Neotropical: Listado de especies con notassobre su biología. Biota Colombiana 3(1):7–118

[CR60] Rausch L et al (2019a) The impact of landscape fragmentation on biodiversity in tropical habitats. Glob Change Biol 25(7):2176–2189

[CR61] Rausch LL, Gibbs HK, Schelly I et al (2019b) Soy expansion in Brazil’s Cerrado. Conserv Lett 12(6):e12671

[CR62] Rebêlo JMM, Moure JS (1995) As espéces de *Euglossa* Latreille do nordeste de São Paulo (Apidae, Euglossinae). Revista Brasileira de Zoologia 12(3):445–466

[CR63] Ribeiro JF, Walter BMT (1998) Fitofisionomias do bioma Cerrado. In: Sano SM, Almeida SP (eds) Cerrado: ambiente e flora. Embrapa Cerrado, Brasília, DF, pp 89–166

[CR65] Rosa JFR, Ramalho M, Monteiro D et al (2015) Permeability of matrices of agricultural crops to Euglossina bees (Hymenoptera, Apidae) in the Atlantic Rain Forest. Apidologie, 46:691–702

[CR64] Rosa JFR, Ramalho M, Arias MC (2016) Functional connectivity and genetic diversity of *Eulaema atleticana* (Apidae, Euglossina) in the Brazilian Atlantic Forest Corridor: assessment of gene flow. Biotropica 48(4):509–517

[CR66] Roubik DW (2004) Sibling Species of *Glossura* and *Glossuropoda* in the Amazon Region (Hymenoptera: Apidae: Euglossini). J Kansas Entomol Soc 77(3):235–253

[CR67] Roubik DW, Hanson PE (2004) Orchid bees of tropical America: biology and field guide. Heredia, Costa Rica. INBio Press. 370:124

[CR68] Sano EE, Rosa R, Brito JLS et al (2010) Land cover mapping of the tropical savanna region in Brazil. Environ Monit Assess 166:113–124 19504057 10.1007/s10661-009-0988-4

[CR69] Saura S (2020) The Habitat Amount Hypothesis implies negative effects of habitat fragmentation on species richness. J Biogeogr 48(1):11–22

[CR73] Silva JM, Bates J (2002) Biogeographic patterns and conservation in the South American Cerrado: A tropical Savanna. Bioscience 52(3):225–233

[CR71] Silva DP, De Marco PJ (2014) No Evidence of Habitat Loss Affecting the Orchid Bees *Eulaema nigrita* Lepeletier and *Eufriesea auriceps* Friese (Apidae: Euglossini) in the Brazilian Cerrado Savanna. Neotrop Entomol 43(6):509–518 27194058 10.1007/s13744-014-0244-7

[CR70] Silva DP, Aguiar AJC, Melo GAR et al (2013) Amazonian species within the Cerrado savanna: new records and potential distribution for *Aglae caerulea* (Apidae: Euglossini). Apidologie 44:673–683

[CR72] Silva DP, Varela S, Nemésio A et al (2015) Adding Biotic Interactions into Paleodistribution Models: A Host–Cleptoparasite Complex of Neotropical Orchid Bees. PLoS ONE 10(6):e0129890 26069956 10.1371/journal.pone.0129890 PMC4466402

[CR74] Sousa FG, Santos JS, Martello F et al (2022) Natural habitat cover and fragmentation per se influence orchid-bee species richness in agricultural landscapes in the Brazilian Cerrado. Apidologie 53:1–13

[CR75] Steffan-Dewenter I, Münzenberg U, Bürger C et al (2002) Scale-Dependent Effects of Landscape Context on Three Pollinator Guilds. Ecology 83(5):1421–1432

[CR76] Storck-Tonon D, Peres CA (2017) Forest patch isolation drives local extinctions of Amazonian orchid bees in a 26 years old archipelago. Biol Conserv 214:270–277

[CR142] Vilhena P dos S, Rocha L de J, Garófalo CA (2017) Male Orchid Bees (Hymenoptera: Apidae: Euglossini) in Canopy and Understory of Amazon Várzea Floodplain Forest. I. Microclimatic, Seasonal and Faunal Aspects. ociobiology 64(2):191–201. 10.13102/sociobiology

[CR77] Wikelski M, Moxley J, Eaton-Mordas A et al (2010) Large-Range Movements of Neotropical Orchid Bees Observed via Radio Telemetry. PLoS ONE 5(5):e10738 20520813 10.1371/journal.pone.0010738 PMC2877081

[CR78] Winfree R, Bartomeus I, Cariveau DP (2011) Native Pollinators in Anthropogenic Habitats. Annu Rev Ecol Evol Syst 42:1–22

[CR79] Winkler K, Fuchs R, Rounsevell M et al (2021) Global land use changes are four times greater than previously estimated. Nat Communicatins 12(2501):1–10 10.1038/s41467-021-22702-2 PMC8113269 33976120

[CR81] Zuur AF, Ieno EN, Walker NJ et al (2009) Mixed Effects Models and Extensions in Ecology with R. Springer, Berlin, Heidelberg

