# Supplementary material for: Orchid bee diversity responds positively to forest cover and landscape heterogeneity in the Brazilian Savanna
Source: Oecologia. 2026 Jun 21;208(7):86. doi: 10.1007/s00442-026-05920-7 (PMC13284026; doi:10.1007/s00442-026-05920-7)
Supplement: Supplementary file 1 — Supplementary Material 1 [file 442_2026_5920_MOESM1_ESM.docx]

**Supplementary Information**

**Table S1.** Sampling sites and Euglossini species richness and abundance in the 20 sampled landscapes in the Brazilian Cerrado. Coordinates are in decimal degrees.

| **Sampling site** | **Coordinates** | | **Observed species richness** | **Estimated species richness** | **Abundance** |
| --- | --- | --- | --- | --- | --- |
|  | **Latitude** | **Longitude** |  |  |  |
| 1 | −16.1851 | −48.6691 | 7 | 8.667 | 26 |
| 2 | −16.6587 | −48.9614 | 6 | 7.667 | 45 |
| 3 | −16.7776 | −48.703 | 4 | 4.833 | 41 |
| 4 | −16.5322 | −48.6961 | 5 | 7.500 | 24 |
| 5 | −16.2326 | −48.7826 | 11 | 16.000 | 32 |
| 6 | −16.9008 | −48.6761 | 4 | 4.833 | 25 |
| 7 | −17.0307 | −48.9377 | 1 | 1.000 | 12 |
| 8 | −16.6373 | −48.6588 | 7 | 8.667 | 60 |
| 9 | −16.4553 | −48.6452 | 3 | 3.833 | 14 |
| 10 | −16.2981 | −48.6598 | 5 | 6.667 | 25 |
| 11 | −16.4801 | −48.3531 | 5 | 5.833 | 23 |
| 12 | −16.7775 | −48.5585 | 7 | 8.667 | 54 |
| 13 | −16.5397 | −48.4998 | 6 | 6.000 | 64 |
| 14 | −16.8748 | −48.1125 | 6 | 7.667 | 63 |
| 15 | −16.8592 | −49.0832 | 5 | 6.667 | 40 |
| 16 | −17.1377 | −48.5361 | 8 | 8.000 | 32 |
| 17 | −16.9107 | −48.3597 | 6 | 8.500 | 20 |
| 18 | −16.9986 | −48.2073 | 7 | 10.333 | 56 |
| 19 | −16.6715 | −48.4831 | 7 | 9.500 | 62 |
| 20 | −16.8602 | −48.4687 | 4 | 4.833 | 10 |

**Table S2.** Sampling sites and Euglossini’s α-diversity in each station of natural (n), natural edge (e), and matrix (m) sampled landscapes in the Brazilian Cerrado.

| **Landscape** | **Site** | ***Aglae caerulea*** | ***Eufriesea auriceps*** | ***Eufriesea cf. mussitans*** | ***Euglossa azurea*** | ***Euglossa cordata*** | ***Euglossa imperialis*** | ***Euglossa leucotricha*** | ***Euglossa melanotricha*** | ***Euglossa pleosticta*** | ***Euglossa securigera*** | ***Euglossa townsendi*** | ***Euglossa truncata*** | ***Eulaema cingulata*** | ***Eulaema nigrita*** |
| --- | --- | --- | --- | --- | --- | --- | --- | --- | --- | --- | --- | --- | --- | --- | --- |
| 1 | n | 0 | 2 | 0 | 0 | 0 | 1 | 0 | 0 | 0 | 2 | 1 | 1 | 1 | 7 |
|  | e | 0 | 1 | 0 | 0 | 0 | 0 | 0 | 0 | 0 | 0 | 0 | 0 | 0 | 1 |
|  | m | 0 | 0 | 0 | 0 | 0 | 0 | 0 | 0 | 0 | 0 | 3 | 1 | 1 | 4 |
| 2 | n | 0 | 1 | 0 | 0 | 0 | 0 | 0 | 0 | 0 | 0 | 4 | 0 | 2 | 13 |
|  | e | 0 | 0 | 0 | 0 | 0 | 1 | 0 | 0 | 0 | 1 | 1 | 0 | 2 | 9 |
|  | m | 0 | 0 | 0 | 0 | 0 | 1 | 0 | 0 | 0 | 0 | 1 | 0 | 1 | 8 |
| 3 | n | 0 | 0 | 0 | 0 | 0 | 13 | 0 | 0 | 0 | 0 | 0 | 0 | 1 | 1 |
|  | e | 0 | 0 | 0 | 1 | 0 | 11 | 0 | 0 | 0 | 0 | 0 | 0 | 1 | 2 |
|  | m | 0 | 0 | 0 | 0 | 0 | 0 | 0 | 0 | 0 | 0 | 0 | 0 | 1 | 10 |
| 4 | n | 0 | 0 | 1 | 0 | 0 | 0 | 0 | 0 | 0 | 0 | 1 | 1 | 0 | 9 |
|  | e | 0 | 0 | 0 | 0 | 0 | 1 | 0 | 0 | 0 | 0 | 0 | 1 | 0 | 4 |
|  | m | 0 | 0 | 0 | 0 | 0 | 0 | 0 | 0 | 0 | 0 | 0 | 0 | 0 | 6 |
| 5 | n | 2 | 1 | 0 | 0 | 0 | 0 | 0 | 0 | 0 | 0 | 0 | 0 | 0 | 2 |
|  | e | 0 | 1 | 0 | 0 | 0 | 0 | 0 | 0 | 0 | 0 | 1 | 1 | 6 | 5 |
|  | m | 0 | 0 | 0 | 0 | 1 | 0 | 1 | 1 | 1 | 1 | 6 | 0 | 1 | 1 |
| 6 | n | 0 | 1 | 0 | 0 | 0 | 0 | 0 | 0 | 0 | 0 | 0 | 0 | 2 | 4 |
|  | e | 0 | 1 | 0 | 0 | 0 | 1 | 0 | 0 | 0 | 0 | 0 | 0 | 1 | 3 |
|  | m | 0 | 2 | 0 | 0 | 0 | 0 | 0 | 0 | 0 | 0 | 0 | 0 | 0 | 10 |
| 7 | n | 0 | 0 | 0 | 0 | 0 | 0 | 0 | 0 | 0 | 0 | 0 | 0 | 0 | 8 |
|  | e | 0 | 0 | 0 | 0 | 0 | 0 | 0 | 0 | 0 | 0 | 0 | 0 | 0 | 0 |
|  | m | 0 | 0 | 0 | 0 | 0 | 0 | 0 | 0 | 0 | 0 | 0 | 0 | 0 | 4 |
| 8 | n | 0 | 2 | 0 | 0 | 0 | 26 | 0 | 0 | 0 | 0 | 1 | 0 | 0 | 9 |
|  | e | 0 | 0 | 0 | 0 | 0 | 0 | 0 | 0 | 1 | 0 | 0 | 0 | 2 | 1 |
|  | m | 0 | 3 | 0 | 0 | 0 | 0 | 0 | 0 | 0 | 1 | 4 | 0 | 3 | 7 |
| 9 | n | 0 | 0 | 0 | 0 | 0 | 0 | 0 | 0 | 0 | 0 | 0 | 0 | 2 | 2 |
|  | e | 0 | 0 | 0 | 0 | 0 | 0 | 0 | 0 | 0 | 0 | 0 | 0 | 0 | 0 |
|  | m | 0 | 2 | 0 | 0 | 0 | 0 | 0 | 0 | 0 | 0 | 0 | 0 | 1 | 7 |
| 10 | n | 0 | 1 | 0 | 0 | 0 | 0 | 0 | 0 | 0 | 1 | 0 | 0 | 0 | 3 |
|  | e | 0 | 1 | 0 | 0 | 0 | 0 | 0 | 0 | 0 | 0 | 0 | 0 | 0 | 0 |
|  | m | 0 | 3 | 0 | 0 | 1 | 0 | 0 | 0 | 0 | 0 | 0 | 0 | 2 | 13 |
| 11 | n | 0 | 0 | 0 | 0 | 0 | 0 | 0 | 0 | 0 | 0 | 0 | 0 | 0 | 6 |
|  | e | 0 | 1 | 0 | 0 | 0 | 0 | 0 | 1 | 0 | 3 | 0 | 0 | 2 | 7 |
|  | m | 0 | 1 | 0 | 0 | 0 | 0 | 0 | 1 | 0 | 0 | 0 | 0 | 1 | 0 |
| 12 | n | 0 | 0 | 0 | 0 | 0 | 5 | 1 | 0 | 0 | 0 | 1 | 0 | 8 | 7 |
|  | e | 0 | 1 | 0 | 1 | 0 | 0 | 0 | 0 | 0 | 0 | 0 | 0 | 9 | 7 |
|  | m | 0 | 0 | 0 | 0 | 0 | 1 | 1 | 0 | 0 | 0 | 2 | 0 | 4 | 6 |
| 13 | n | 0 | 3 | 0 | 0 | 0 | 0 | 0 | 0 | 0 | 2 | 5 | 1 | 4 | 10 |
|  | e | 0 | 2 | 0 | 0 | 0 | 0 | 0 | 0 | 0 | 2 | 0 | 1 | 2 | 10 |
|  | m | 0 | 4 | 0 | 0 | 0 | 0 | 0 | 0 | 0 | 5 | 8 | 1 | 0 | 4 |
| 14 | n | 0 | 9 | 0 | 0 | 0 | 5 | 0 | 0 | 0 | 1 | 0 | 0 | 0 | 16 |
|  | e | 0 | 11 | 0 | 0 | 0 | 4 | 0 | 0 | 0 | 1 | 1 | 0 | 0 | 6 |
|  | m | 0 | 0 | 0 | 0 | 0 | 1 | 0 | 0 | 0 | 0 | 0 | 0 | 1 | 7 |
| 15 | n | 0 | 1 | 0 | 0 | 0 | 1 | 0 | 0 | 0 | 0 | 0 | 0 | 1 | 8 |
|  | e | 0 | 0 | 0 | 1 | 0 | 0 | 0 | 0 | 0 | 0 | 0 | 0 | 2 | 1 |
|  | m | 0 | 4 | 0 | 0 | 0 | 0 | 0 | 0 | 0 | 0 | 0 | 0 | 1 | 20 |
| 16 | n | 0 | 0 | 0 | 0 | 0 | 1 | 0 | 1 | 0 | 0 | 1 | 0 | 4 | 6 |
|  | e | 0 | 1 | 0 | 0 | 0 | 5 | 0 | 1 | 0 | 1 | 1 | 1 | 1 | 0 |
|  | m | 0 | 1 | 0 | 0 | 0 | 0 | 0 | 1 | 0 | 1 | 0 | 1 | 1 | 3 |
| 17 | n | 0 | 1 | 0 | 0 | 0 | 0 | 0 | 0 | 0 | 0 | 0 | 0 | 3 | 9 |
|  | e | 0 | 1 | 0 | 0 | 0 | 1 | 0 | 0 | 0 | 0 | 1 | 0 | 0 | 0 |
|  | m | 0 | 0 | 0 | 0 | 0 | 0 | 0 | 1 | 0 | 0 | 0 | 0 | 0 | 3 |
| 18 | n | 0 | 2 | 0 | 0 | 0 | 7 | 0 | 1 | 0 | 1 | 1 | 0 | 0 | 14 |
|  | e | 0 | 4 | 0 | 0 | 0 | 0 | 0 | 0 | 0 | 0 | 0 | 0 | 2 | 9 |
|  | m | 0 | 2 | 0 | 0 | 0 | 0 | 0 | 1 | 0 | 0 | 0 | 0 | 0 | 12 |
| 19 | n | 0 | 1 | 0 | 0 | 0 | 2 | 0 | 0 | 0 | 2 | 1 | 0 | 1 | 10 |
|  | e | 0 | 0 | 0 | 0 | 0 | 3 | 0 | 0 | 0 | 0 | 0 | 1 | 2 | 14 |
|  | m | 0 | 4 | 0 | 0 | 0 | 0 | 0 | 0 | 0 | 0 | 0 | 0 | 1 | 20 |
| 20 | n | 0 | 0 | 0 | 0 | 0 | 0 | 0 | 0 | 0 | 0 | 0 | 0 | 1 | 2 |
|  | e | 0 | 0 | 0 | 0 | 0 | 1 | 0 | 0 | 0 | 0 | 0 | 0 | 1 | 0 |
|  | m | 0 | 0 | 0 | 0 | 0 | 0 | 0 | 0 | 0 | 0 | 0 | 2 | 1 | 2 |
|  | **Total** | **2** | **76** | **1** | **3** | **2** | **92** | **3** | **9** | **2** | **25** | **45** | **13** | **83** | **372** |

**Table S3.** Spatial autocorrelation of the species richness and abundance of Euglossini bees using the Moran I test. SD, standard deviation.

| **Response variable** | **Observed** | **Expected** | **SD** | **p** |
| --- | --- | --- | --- | --- |
| Species richness | −0.028 | −0.052 | 0.036 | 0.506 |
| Abundance | −0.025 | −0.052 | 0.041 | 0.508 |

**Table S4.** The scale of effect for Euglossini bee species richness, abundance, and species composition. The table shows the coefficients of determination (R^2^) for each response variable and landscape metrics. Significant p-values (p < 0.05) are in bold. The predictor variables estimated were FC, forest cover; SC, savanna cover; AC, agriculture cover; PC, pasture cover; CH, compositional heterogeneity; SH, shape mean index.

|  | **Estimated Species Richness** | | | | | **Abundance** | | | | | **Species Composition** | | | | |
| --- | --- | --- | --- | --- | --- | --- | --- | --- | --- | --- | --- | --- | --- | --- | --- |
| **Landscape metric** | **500 m** | **1000 m** | **1500 m** | **2000 m** | **3000 m** | **500 m** | **1000 m** | **1500 m** | **2000 m** | **3000 m** | **500 m** | **1000 m** | **1500 m** | **2000 m** | **3000 m** |
| **FC** | 0.002 | 0.003 | 0.010 | 0.035 | **0.042** | 0.022 | 0.060 | 0.064 | 0.083 | **0.088** | 0.010 | 0.008 | 0.0134 | 0.011 | **0.026** |
| **SC** | 0.004 | 0.002 | **0.005** | 0.002 | 0.003 | 0.018 | **0.036** | 0.034 | 0.014 | 0.006 | 0.041 | 0.046 | 0.082 | 0.099 | **0.115** |
| **AC** | 0.013 | 0.028 | 0.096 | **0.132** | 0.118 | **0.172** | 0.162 | 0.155 | 0.150 | 0.121 | 0.0186 | 0.044 | 0.139 | **0.173** | 0.170 |
| **PC** | 0.003 | 0.005 | 0.052 | **0.091** | 0.071 | 0.001 | 0.001 | 0.029 | 0.076 | **0.087** | **0.026** | 0.012 | 0.006 | 0.015 | 0.009 |
| **CH** | 0.005 | 0.000 | 0.058 | 0.052 | **0.058** | 0.001 | 0.018 | **0.147** | 0.121 | 0.070 | **0.125** | 0.021 | 0.063 | 0.117 | 0.109 |
| **SH** | **0.028** | 0.010 | 0.005 | < 0.001 | 0.012 | 0.040 | < 0.001 | **0.067** | 0.002 | 0.005 | 0.005 | 0.0005 | 0.015 | 0.006 | **0.040** |

**Table S5.** Analysis of multicollinearity among explanatory variables based on the variance inflation factor (VIF) for species richness and abundance. FC, forest cover; SC, savanna cover; AC, agriculture cover; PC, pasture cover; CH, compositional heterogeneity; SH, shape mean index.

| **Landscape metric** | **Estimate** | **SE** | **t** | **p** | **VIF** |
| --- | --- | --- | --- | --- | --- |
| **Estimated Species Richness** | | | | | |
| Intercept | 8.80 | 23.28 | 0.38 | 0.71 |  |
| FC (3000 m) | -0.02 | 0.24 | -0.10 | 0.92 | 6.39 |
| SC (500 m) | -0.03 | 0.18 | -0.19 | 0.85 | 26.28 |
| PC (2000 m) | 0.01 | 0.20 | 0.06 | 0.95 | 21.65 |
| AC (2000 m) | -0.06 | 0.18 | -0.31 | 0.76 | 32.80 |
| CH (3000 m) | 3.75 | 4.98 | 0.75 | 0.46 | 1.90 |
| SH (500 m) | -2.68 | 2.92 | -0.92 | 0.37 | 1.70 |
| Variables with VIF<3 |  |  |  |  |  |
| Intercept | 1.62 | 6.70 | 0.24 | 0.81 |  |
| FC (3000 m) | 0.04 | 0.13 | 0.31 | 0.76 | 2.10 |
| SC (500 m) | 0.01 | 0.05 | 0.17 | 0.86 | 2.56 |
| PC (2000 m) | 0.06 | 0.07 | 0.84 | 0.42 | 2.95 |
| CH (3000 m) | 3.71 | 4.84 | 0.77 | 0.46 | 1.87 |
| SH (500 m) | −1.46 | 2.38 | −0.61 | 0.55 | 1.17 |
| **Abundance** | | | | | |
| Intercept | 2.07 | 0.67 | 3.06 | 0.00 |  |
| FC (3000 m) | 0.01 | 0.01 | 0.63 | 0.53 | 3.00 |
| SC (1000 m) | -0.01 | 0.00 | -1.45 | 0.15 | 6.21 |
| AC (500 m) | -0.01 | 0.00 | -3.42 | 0.00 | 3.38 |
| PC (3000 m) | 0.01 | 0.00 | 1.20 | 0.23 | 3.38 |
| CH (1500 m) | 1.40 | 0.29 | 4.80 | 0.00 | 2.21 |
| SH (1500 m) | -0.12 | 0.30 | -0.41 | 0.68 | 1.29 |
| Variables with VIF<3 |  |  |  |  |  |
| Intercept | 3.09 | 43.49 | 0.07 | 0.94 |  |
| FC (3000 m) | 0.49 | 0.86 | 0.57 | 0.58 | 2.92 |
| AC (500 m) | −0.36 | 0.25 | −1.44 | 0.18 | 1.34 |
| PC (3000 m) | 0.39 | 0.42 | 0.92 | 0.38 | 2.98 |
| CH (1500 m) | 46.63 | 26.97 | 1.73 | 0.11 | 1.82 |
| SH (1500 m) | −14.27 | 24.63 | −0.58 | 0.57 | 1.87 |
